# Supplementary material for: TabDEG: Classifying differentially expressed genes from RNA-seq data based on feature extraction and deep learning framework
Source: PLoS One. 2024 Jul 22;19(7):e0305857. doi: 10.1371/journal.pone.0305857 (PMC11262683; doi:10.1371/journal.pone.0305857)
Supplement: S1 File — (PDF) [file pone.0305857.s001.pdf]

Supplement

I. ROC curves with scores for different methods across ten datasets

BRCA

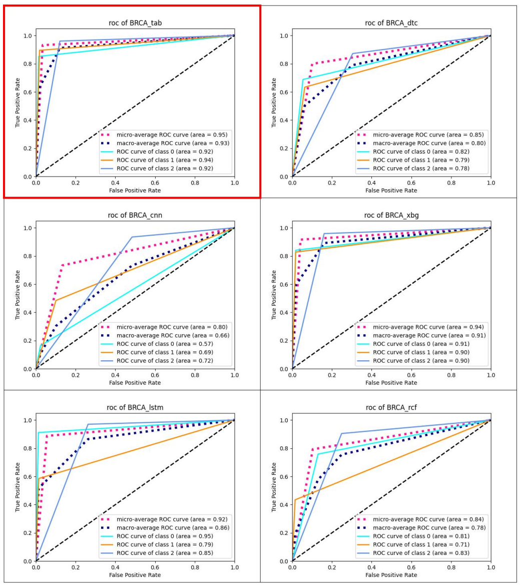

COAD

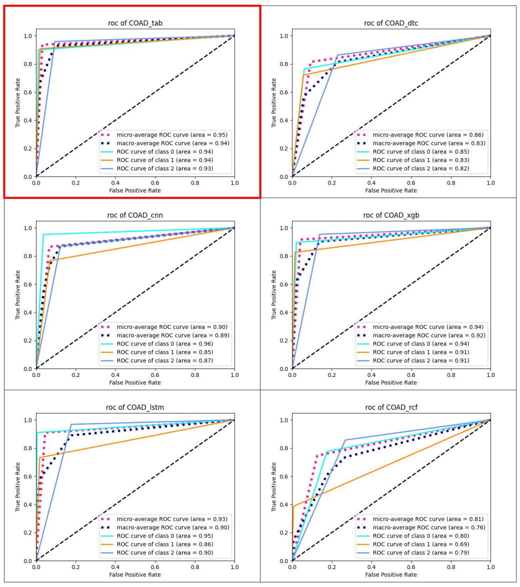

HNSC

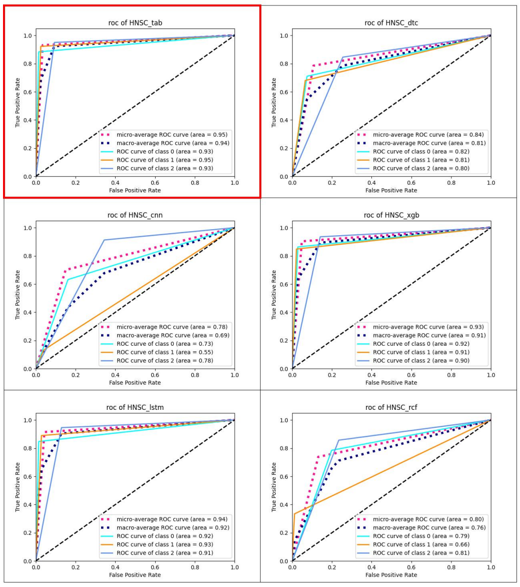

KIRC

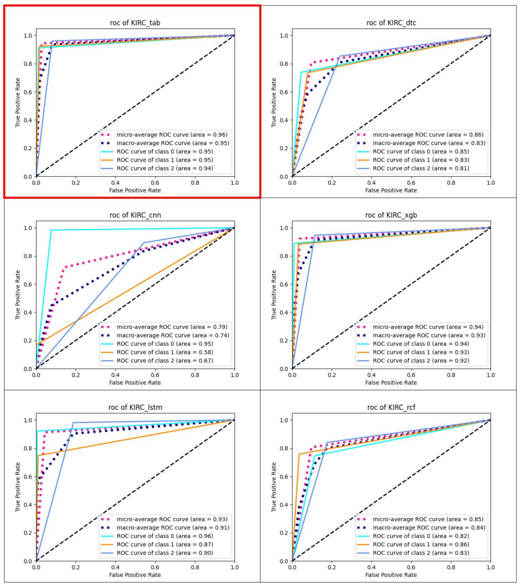

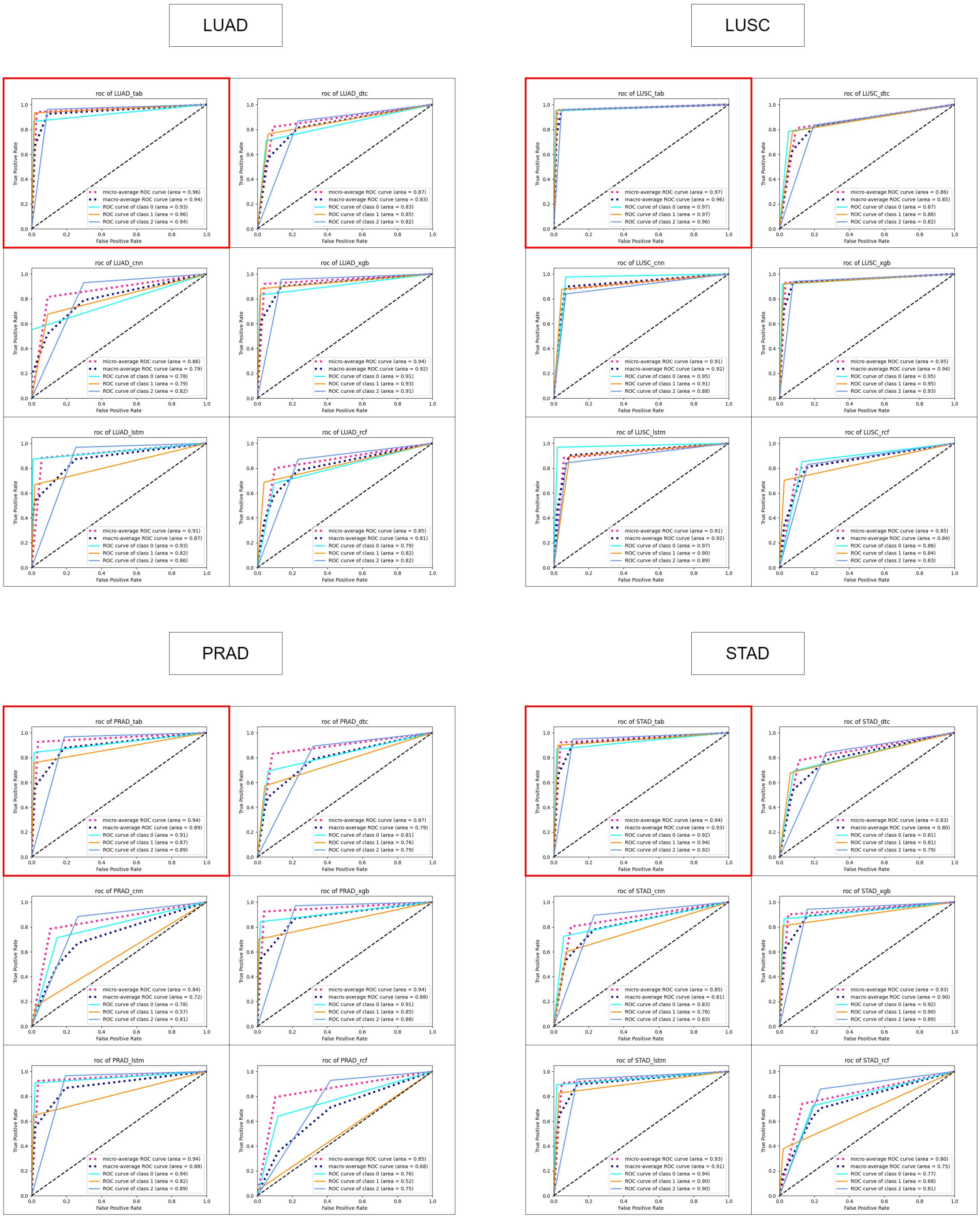
LUAD
LUSC
roc of LUAD_tab
roc of LUAD_dtc
roc of LUAD_cnn
roc of LUAD_xgb
roc of LUAD_lstm
roc of LUAD_rcf
roc of LUSC_tab
roc of LUSC_dtc
roc of LUSC_cnn
roc of LUSC_xgb
roc of LUSC_lstm
roc of LUSC_rcf
PRAD
STAD
roc of PRAD_tab
roc of PRAD_dtc
roc of PRAD_cnn
roc of PRAD_xgb
roc of PRAD_lstm
roc of PRAD_rcf
roc of STAD_tab
roc of STAD_dtc
roc of STAD_cnn
roc of STAD_xgb
roc of STAD_lstm
roc of STAD_rcf

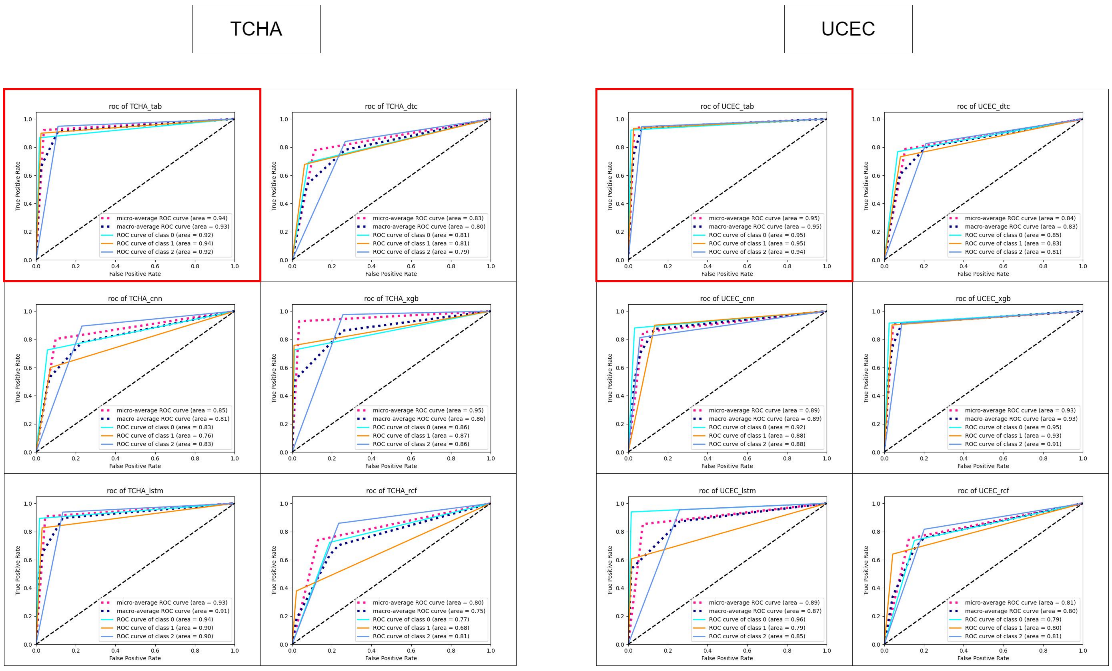
TCHA
UCEC
roc of TCHA_tab
roc of TCHA_dtc
roc of TCHA_cnn
roc of TCHA_xgb
roc of TCHA_lstm
roc of TCHA_rcf
roc of UCEC_tab
roc of UCEC_dtc
roc of UCEC_cnn
roc of UCEC_xgb
roc of UCEC_lstm
roc of UCEC_rcf
