# Supplementary material for: TabDEG: Classifying differentially expressed genes from RNA-seq data based on feature extraction and deep learning framework
Source: PLoS One. 2024 Jul 22;19(7):e0305857. doi: 10.1371/journal.pone.0305857 (PMC11262683; doi:10.1371/journal.pone.0305857)
Supplement: S2 File — (PDF) [file pone.0305857.s002.pdf]

Supplement

II. : Mapped predicted genes in cancer pathways

We performed pathway enrichment analysis on the predicted UR and DR genes in the biological test data of the BRCA and UCEC datasets. In the Figure, we report 13 important pathways related to the progress of various cancer datasets, all of which are sourced from the predicted UR and DR genes of BRCA and UCEC. We will discuss a few genes mapped to these pathways in the following paragraphs.

BRCA Datasets: In general, BRCA gene mutations can affect multiple signaling pathways and physiological pro cesses, including but not limited to DNA repair, cell cycle regulation, apoptosis, transcription regulation, protein stability, and signal transduction. WP OXIDATIVE STRESS RESPONSE: This pathway involves the organism's response and regulation to oxidative stress. The production of oxygen free radicals and other reactive substances can cause DNA damage, accumulation of oxidized proteins and lipids, and lead to various diseases, including cancer. In the process of cancer initiation and progression, the level of oxidative stress in the body is often affected, resulting in physiological effects such as DNA damage and abnormal cell cycle, thereby promoting the growth and metastasis of tumor cells. For example, the FOS gene encodes a transcription factor protein, the c-Fos precursor protein, which plays an important role in multiple biological processes such as cell proliferation, differentiation, apoptosis, and inflammation. Abnormal expression of this gene is related to the occur rence and development of breast cancer.

UCEC Datasets: Studies have shown that the molecular composition associated with the tumor microenvironment in endometrial cancer tissue includes genes related to neurodevelopment and angiogenesis, as well as extracellular matrix proteins related to the interaction between tumor cells and the matrix (including components in NABA CORE MATRISOME). For example, HGF encodes a promoter, which is an important factor that promotes cell proliferation and migration and is closely related to the occurrence and development of UCEC cancer. IGFBP6 encodes an extracel lular matrix protein that can inhibit apoptosis and promote cell proliferation, and has been shown to be upregulated in UCEC cell lines.

Cancer	Pathways	Mapped predicted genes
UCEC_UP	KEGG_SYSTEMIC_LUPUS_ERYTHEMATOSUS	HLA-DQB1,FCGR3B,C1QC,C2
	REACTOME_TRANSCRIPTIONAL_REGULATION_OF_GRANULOPOIESIS	SPI1
	REACTOME_ANTIMICROBIAL_PEPTIDES	BPIFA1,S100A7,SLC11A1,BPIFB4,BPIFB2, PRSS3,RNASE7,BPIFB6,LCN2
	REACTOME_TRANSCRIPTIONAL_REGULATION_BY_SMALL_RNAS	POLR2I,NUP210
UCEC_DOWN	NABA_MATRISOME	HGF,SERPINF1,CD109,CCL26,FGF7,CCL15,COL6A3,VTN,MMP28,WNT2B, WNT9B,NRG2,IGFBP5,IGFBP6,CLEC14A,GAS6,SEMA3G,FREM1,FGF16, SULF2,NGF,CHRDL1,NID1,ADAMTSL1,LAMA4,EMILIN1,TIMP2,CCBE1, TLL1,EGFL7,ANGPTL7,LGI1
	KEGG_NEUROACTIVE_LIGAND_RECEPTOR_INTERACTION	TSHB,ADRA1A,ADRB3,HTR1B,FSHR,GABBR1,AVPR1A,AVPR2,PTGER3, PTH1R,TACR1,NMBR,NPY1R,GRID1,GRIN2A,S1PR3,EDNRA,P2RX1,LPAR6
	NABA_CORE_MATRISOME	CRIM1,HMCN2,COL6A3,FBLN5,VTN,LGI4,IGFBP5,IGFBP6,PRELP,LGI3,PODN, GAS6,COL21A1,NID1,IMPG2,LAMA4,KERA,EMILIN1,LGI2,LGI1
BRCA_UP	NABA_MATRISOME	REG4, HPSE2, S100A2, S100A8, S100A12, SERPINB5, MMP9, CCL11, CCL19, CLEC10A, CCL24, MMP26, COL2A1, AMBP, PAPPA2, COL4A6, COL9A1, COL11A2, ITLN1, BCAN, EGFL6, S100A7A, SCUBE2, SLIT1, IL1A, MUC7, FBN3, ADAM18, CST11, GDF1, NELL1, BMP7
	NABA_MATRISOME_ASSOCIATED	REG4,HPSE2,S100A2,S100A8,S100A12,SERPINB5,MMP9,CCL11,CCL19,CLEC10A, CCL24,MMP26,AMBP,ITLN1,MUC13,S100A7A,SCUBE2,IFNE,IL1A,MUC7,IFNK, BRINP3,ADAM18,IL17C,CST11,GDF1,BMP7,ADAMDEC1
	KEGG_METABOLISM_OF_XENOBIOTICS_BY_CYTOCHROME_P450	UGT2B4,UGT2B28,UGT1A10,CYP2B6,CYP2F1,GSTA1,GSTM5,UGT2A3
BRCA_DOWN	KEGG_FOCAL_ADHESION	COMP,COL6A6,ACTN3,CAV3,ITGA7,THBS4,IGF1,PAK3,MAPK10,VEGFD,TNXB
	NABA_CORE_MATRISOME	DPT,GLDN,FMOD,COMP,COL6A6,MMRN1,THBS4,ADIPOQ,PCOLCE2,TNXB
	WP_OXIDATIVE_STRESS_RESPONSE	MAOA,FOS,MAPK10,SOD3
