# Supplementary material for: TabDEG: Classifying differentially expressed genes from RNA-seq data based on feature extraction and deep learning framework
Source: PLoS One. 2024 Jul 22;19(7):e0305857. doi: 10.1371/journal.pone.0305857 (PMC11262683; doi:10.1371/journal.pone.0305857)
Supplement: Attachment — Submitted filename: review.docx [file pone.0305857.s003.docx]

The work is interesting. This paper designs framework for predicting DEGs. Although the promising results have been achieved, several major concerns in the current manuscript show that it appears that publication in any form would be premature at this time.

1. The relationship between the data downloaded from UCSX Xena Data Browser (https : //xenabrowser.net/datapages/) and the data of TCGA should be listed.

2.” Import the ”tidyverse” package and then start pre-processing the gene expression

data. RNA transcripts with an expression level greater than 1 are screened out and a

total of 17983 RNA transcripts are screened. The screen-obtained datasets are divided into T and N, which are used as control conditions in next step.” Here, how to divide the two datasets ”T” and “N”?

3. The readability of writing is poor, which makes the manuscript is difficult to be understood.

4. Tables 3 and 4 can be combined and shown graphically.

5. Including the benchmarking algorithms, what is the input of the algorithms? What kind of feature extraction method is used?
