# Supplementary material for: TabDEG: Classifying differentially expressed genes from RNA-seq data based on feature extraction and deep learning framework
Source: PLoS One. 2024 Jul 22;19(7):e0305857. doi: 10.1371/journal.pone.0305857 (PMC11262683; doi:10.1371/journal.pone.0305857)
Supplement: Attachment — Submitted filename: Response to Reviewers.docx [file pone.0305857.s004.docx]

Dear Editors and Reviewers:

Thank you for your letter and for the reviewers’ comments concerning our manuscript entitled “TabDEG: Classifying differentially expressed genes from RNA-seq data based on feature extraction and deep learning framework” (PONE-D-23-30756). Those comments are all valuable and very helpful for revising and improving our paper, as well as the important guiding significance to our researches. We have studied comments carefully and have made correction which we hope meet with approval. The maincorrections in the paper and the responds to the reviewer's comments are as flowing:

Responds to the reviewer's comments:

Reviewer#1:

1.Response to comment: The authors should mention in detail how they selected the 10 different tumor types characteristic (Age, gender, stage, TNM, et al) for the experiment and discuss the potential bias of the results.

Response:

The TCGA dataset selected in this paper comes from a variety of samples from patients with different regions, races and clinical characteristics, and thus contains different genomic information, which helps to reduce group bias and improve the representativeness of the results. Meanwhile, different tumor types may have different characteristics, development patterns, and treatments, so by using datasets of multiple tumor types, the over-reliance on specific tumor types can be reduced, and the generalization ability of the algorithm can be improved, making it more robust in dealing with unknown or novel tumor types. Relevant revisions we have marked in yellow color in the "Data Collection and Preprocessing" section of the revised manuscript.

2.Response to comment:The author should justify using logfc > 1 as a threshold for DEGs identification.

Responsc:

LogFC > 1 indicates at least a 2-fold change in the expression level of a gene between two conditions, and such a change is considered to be relatively large to provide a higher significance difference and thus more likely to reflect biological importance. In many related studies, logFC > 1 is considered a reasonable threshold for screening out DEGs of functional and biological importance. Relevant revisions we have marked in yellow color in the "Data Collection and Preprocessing" section of the revised manuscript.

3.Response to comment:Necessary references should be added, such as the section on "Path enrich analysis of predicted UR and DR genes".

Responsc:

We sincerely appreciate the valuable comments. We have checked the literature carefully and added more references into the section on "Path enrich analysis of predicted UR and DR genes" part in the revised manuscript.

4.Response to comment:English needs to be improved by English editing service or a native English speaker.

Responsc:

Thanks for your suggestion. We have tried our best to polish the language in the revised manuscript.

Reviewer#2:

1.Response to comment: The relationship between the data downloaded from UCSX Xena Data Browser (https : //xenabrowser.net/datapages/) and the data of TCGA should be listed.

Response:

The UCSX Xena Data Browser serves as a data browser that provides access to and analyzes TCGA data. Relevant revisions we have marked in red color in the "Data Collection and Preprocessing" section of the revised manuscript.

2.Response to comment: ” Import the ”tidyverse” package and then start pre-processing the gene expression data. RNA transcripts with an expression level greater than 1 are screened out and a

total of 17983 RNA transcripts are screened. The screen-obtained datasets are divided into T and N, which are used as control conditions in next step.” Here, how to divide the two datasets ”T” and “N”?

Response:

The tumor and normal groups will be labeled "01A" and "11A" respectively in the column names of the TCGA dataset, and based on this the dataset will be categorized into "T" and "N" groups. Relevant revisions we have marked in red color in the "Data Collection and Preprocessing" section of the revised manuscript.

3.Response to comment: The readability of writing is poor, which makes the manuscript is difficult to be understood.

Response:

Thanks for your suggestion. We have tried our best to polish the language in the revised manuscript.

4.Response to comment: Tables 3 and 4 can be combined and shown graphically.

Response:

We have completed the relevant revisions in the Table 3 of the revised manuscript.

5.Response to comment: Including the benchmarking algorithms, what is the input of the algorithms? What kind of feature extraction method is used?

Response:

The input to the algorithm is RNA-seq counts data, and the feature extraction uses a combination of pca, k-means and umap for dimensionality reduction extraction.

6.Response to comment: It was suggested that authors should share the code and data in this work.

Response:

We will share the code and data from this work in the Supporting Information.

Special thanks to you for your good comments.
