# Supplementary material for: TabDEG: Classifying differentially expressed genes from RNA-seq data based on feature extraction and deep learning framework
Source: PLoS One. 2024 Jul 22;19(7):e0305857. doi: 10.1371/journal.pone.0305857 (PMC11262683; doi:10.1371/journal.pone.0305857)
Supplement: Attachment — Submitted filename: Response to Reviewers2.docx [file pone.0305857.s005.docx]

Dear Editors and Reviewers:

Thank you for your letter and for the reviewers’ comments concerning our manuscript entitled “TabDEG: Classifying differentially expressed genes from RNA-seq data based on feature extraction and deep learning framework” (PONE-D-23-30756R1). Those comments are all valuable and very helpful for revising and improving our paper, as well as the important guiding significance to our researches. We have studied comments carefully and have made correction which we hope meet with approval. The maincorrections in the paper and the responds to the reviewer's comments are as flowing:

Responds to the reviewer's comments:

Reviewer#2:

Authors should share the code and data in this work，it can be posted on GitHub instead of screenshots in supplementary materials

Response:

Thanks for the correction, we have posted the code and data in this work on GitHub, which can be viewed at the link: <https://github.com/xueyupi/my_tabdeg>

Special thanks to you for your good comments.
